# Neuroprotection Effect of Astragaloside IV from 2-DG-Induced Endoplasmic Reticulum Stress

**DOI:** 10.1155/2020/9782062

**Published:** 2020-12-31

**Authors:** Yu Fu, Jianhang Cai, Mengyao Xi, Yifei He, Yang Zhao, Yi Zheng, Yidong Zhang, Jinkun Xi, Yonggui He

**Affiliations:** ^1^Clinic School of Medicine and Affiliated Hospital, North China University of Science and Technology, Tangshan 063000, China; ^2^Hebei Key Laboratory for Chronic Diseases, North China University of Science and Technology, Tangshan 063000, China; ^3^Department of Neurology, Dongming People's Hospital, Dongming 274501, China; ^4^School of Nursing, Dalian Medical University, Dalian 116044, China

## Abstract

**Objective:**

Astragaloside IV shows neuroprotective activity, but its mechanism remains unclear. To investigate whether astragaloside IV protects from endoplasmic reticulum stress (ERS), we focus on the regulation of glycogen synthase kinase-3*β* (GSK-3*β*) and mitochondrial permeability transition pore (mPTP) by astragaloside IV in neuronal cell PC12.

**Methods and Results:**

PC12 cells treated with different concentrations of ERS inductor 2-deoxyglucose (2-DG) (25-500 *μ*M) showed a significant increase of glucose-regulated protein 78 (GRP 78) and GRP 94 expressions and a decrease of tetramethylrhodamine ethyl ester (TMRE) fluorescence intensity and mitochondrial membrane potential (∆*Ψ*m), with the peak effect seen at 50 *μ*M, indicating that 2-DG induces ERS and the mPTP opening. Similarly, 50 *μ*M of astragaloside IV increased the GSK-3*β* phosphorylation at Ser9 most significantly. Next, we examined the neuroprotection of astragaloside IV by dividing the PC12 cells into control group, 2-DG treatment group, astragaloside IV plus 2-DG treatment group, and astragaloside IV only group. PC12 cells treated with 50 *μ*M 2-DG for different time courses (0-36 hr) showed a significant increase of Cleaved-Caspase-3 with the peak at 6 hr. 2-DG significantly induced cell apoptosis and increased the green fluorescence intensity of Annexin V-FITC, and these effects were reversed by astragaloside IV. Such a result indicates that astragaloside IV protected neural cell survival from ERS. 2-DG treatment significantly increased the expressions of inositol-requiring ER-to-nucleus signal kinase 1 (IRE1), phosphor-protein kinase R-like ER kinase (p-PERK), but not affect the transcription factor 6 (ATF6) expression. 2-DG treatment significantly decreased the phosphorylation of GSK-3*β* and significantly reduced the TMRE fluorescence intensity and ∆*Ψ*m, following mPTP open. Astragaloside IV significantly inhibited the above effects caused by 2-DG, except the upregulation of ATF6 protein. Taken together, astragaloside IV significantly inhibited the ERS caused by 2-DG.

**Conclusion:**

Our data suggested that astragaloside IV protects PC12 cells from ERS by inactivation of GSK-3*β* and preventing the mPTP opening. The GRP 78, GRP 94, IRE1, and PERK signaling pathways but not ATF6 are responsible for GSK-3*β* inactivation and neuroprotection by astragaloside IV.

## 1. Introduction

Astragaloside IV is the most active saponin compound with a molecular formula C41H68O14. It is soluble in highly polar organic solution [1, 2]. Previous studies have shown protective effects of astragaloside IV on various organs such as heart [3, 4], kidney [5, 6], brain [7, 8], and nerve [9–11].

Endoplasmic reticulum (ER) is an important organelle folding cell surface and secreted protein in eukaryotic cells [12]. Imbalance of ER steady state leads to ERS [13] with the hall markers GRP 78 and GRP 94, which participates in nerve damage [14–16].

Mitochondria are the energy warehouse of eukaryotic cells [17]. Mitochondria are vital in nerve damage [18, 19], which opens mPTP on the mitochondrial membrane and leads to the destruction of mitochondrial structure and cell damage [20–22]. Cross-talk between the endoplasmic reticulum and mitochondria are critical to resist apoptosis and cell stress [23, 24]. GSK-3*β* controls cell growth, survival, and neuronal plasticity in the nervous system [25, 26]. Inactivation of GSK-3*β* to prevent mPTP opening is vital for neuroprotection [18].

ERS causes the accumulation of unfolded or misfolded proteins in the ER, triggering unfolded protein response (UPR) [27]. PERK, IRE1, and ATF6 are the molecular markers of three signaling pathways of ERS. UPR activated IRE1 and damages endothelial cells [28, 29]. PERK and ATF6 pathways participate in the protection of the ischemic brain [30, 31].

Astragaloside IV is a potent neuroprotective saponin compound, which reduces ERS, but the mechanism remains unclear. This study tested whether astragaloside IV inhibits ERS by inactivating GSK-3*β* and preventing mPTP from opening.

## 2. Material and Methods

### 2.1. Cell Culture

Rat adrenal pheochromocytoma-derived PC12 cell was purchased from Baoshide (Wuhan, China). Cells were cultured in Dulbecco's modified Eagle's medium (DMEM) supplemented with 10% fetal bovine serum (FBS) (Invitrogen) and 100 U penicillin/streptomycin at 37°C in a humidified 5% CO_2_-95% air atmosphere. Culture medium changed every 2 to 3 days. Cells were passaged using 0.25% trypsin when cell density reaches about 90%.

### 2.2. Reagents

Following chemicals were used in this study: astragaloside IV (cat. no. HQJG-20110507, Tianjin Mark Biotechnology Co.), 2-Deoxy-D-glucose (cat. no. D6134, Sigma), Tetramethylrhodamine ethylester (cat. no. T669, Molecular Probes), Annexin V-FITC Apoptosis Detection kit (cat. no. C1062 L, Beyotime Biotechnology), Cell culture reagents DMEM (cat. no. C11995500BT), FBS (cat. no. 16140071), 0.25% trypsin-EDTA (cat. no. 25200-056), and penicillin/streptomycin (cat. no. 15140-122) were purchased from Gibco BRL.

All rabbit antibodies were obtained from Cell Signaling Technology Inc. Monoclonal antibody against: GRP 78 (cat. no. 3177), GRP 94 (cat. no. 20292), p-PERK (cat. no. 3179), ATF6 (cat. no. 65880), IRE1 (cat. no. 3294), p-GSK-3*β* (cat. no. 9323), GSK-3*β* (cat. no. 12456), and GADPH (cat. no. 5174). Polyclonal antibody against: Cleaved Caspase-3 (cat. no. 9661), Caspase-3 (cat. no. 9662), and anti-rabbit IgG (cat.no. 14708). All antibodies were used at 1 : 1,000 dilution.

### 2.3. Drug Treatment Group

PC12 cells were grown to 90% confluence and treated as follows. To determine the optimal concentration of 2-DG, PC12 cells were treated with 25, 50, 100, 150, 200, and 500 *μ*M 2-DG or vehicle only for 30 min. To determine the optimal concentration of astragaloside IV, PC12 cells were treated with 25, 50, 75, and 100 *μ*M astragaloside IV or vehicle only for 20 min.

To observe the effect of astragaloside IV on ERS, PC12 cells were divided into 4 groups: (1) control group: treat with vehicle only; (2) 2-DG group: treat with 50 *μ*M 2-DG for 30 min; (3) astragaloside IV+2-DG group: treat with 50 *μ*M astragaloside IV for 20 min then 50 *μ*M 2-DG for 30 min; (4) astragaloside IV group: treat with 50 *μ*M astragaloside IV for 20 min.

### 2.4. Drug Treatment Schedule

PC12 cell culture was washed twice with PBS and then incubated in Tyrode solution for 2 hours prior to other treatments. To examine the effect of astragaloside IV on protein expressions of GRP 78, GRP 94, IRE1, p-PERK, ATF6, p-GSK-3*β*, and GSK-3*β*, PC12 cells were exposed to 2-DG (50 *μ*M) for 30 min. Astragaloside IV (50 *μ*M) was applied 20 min before exposure to 2-DG. PC12 cells were exposed to 50 *μ*M 2-DG for 30 min before *ΔΨ*m measurement and 6 hr before apoptosis measurement, respectively.

### 2.5. Western Blotting

After drug treatment, PC12 cells were washed twice with PBS, and cell pellets were lysed on ice for 30 min with 50 *μ*L of fresh cell lysis buffer (Cell Signaling Technology, cat. no. 9803). The lysate was ultrasonicated and then centrifuged at 12 000 r (4°C) for 15 min. Protein concentration was measured using a BCA kit and then divided into equal protein amount for boiling and loading. After electrophoresis, protein was transferred to PVDF membrane and incubated in 10% nonfat milk at room temperature for 90 min. Blot membranes were incubated with primary antibodies against GRP 78, GRP 94, IRE1, p-PERK, ATF6, p-GSK-3*β*, and GSK-3*β* overnight at 4°C. After incubating the secondary antibody at room temperature for 1 h, ECL fluorescence was developed and the blot image was captured and analyzed with Biospectrum Imaging System (UVP, Upland, USA).

### 2.6. Confocal Imaging of Mitochondrial Membrane Potential

Mitochondrial membrane potential (*ΔΨm*) was measured by loading PC12 cells with cell permeable, cationic mitochondria probe TMRE, as reported previously [32]. TMRE is accumulated specifically by the mitochondria in proportion to mitochondrial membrane potential (∆*Ψ*m) [33]. Loss of *ΔΨ*m is caused by the mPTP opening [34]. Briefly, PC12 cell culture in a temperature-controlled culture dish (MatTek, MA, USA) was incubated with TMRE (100 nM) in standard Tyrode solution containing (mM) NaCl 140, KCl 6, MgCl_2_ 1, CaCl_2_ 1, HEPES 5, and glucose 5.8 (pH 7.4) for 20 minutes. Cells were washed several times with fresh Tyrode solution. The dish was then mounted on the stage of an Olympus FLUOVIEW FV-1000 laser scanning confocal microscope with a 543 nm excitation (Olympus Corporation, Tokyo, Japan). Fluorescence intensity of 10 random-picked cells in each field was measured by FLUOVIEW FV-1000 software at 0 min and measured the average fluorescence intensity value at 0 and 30 min. The changes of fluorescence intensity were expressed as the ratio of average fluorescence intensity of the 10 picked cells at 30-minute over that at 0-minute.

### 2.7. Confocal Imaging of Apoptosis

Apoptosis of PC12 cells was measured using Annexin V-FITC Apoptosis Detection kit following the manual. Briefly, cell culture in a temperature-controlled culture dish (MatTek, MA, USA) was incubated with Annexin V-FITC and PI (Propidium Iodide) for 20 minutes with binding solution at RT in the dark and was washed 3 times with PBS according to the manual. The dish was then mounted on the stage of an Olympus FLUOVIEW FV-1000 laser scanning confocal microscope with 488 and 543 nm excitation (Olympus Corporation, Tokyo, Japan). Quantitative analysis of fluorescence intensity changes by FLUOVIEW FV-1000 -software.

### 2.8. Cell Survival Analysis

Cell survival was assessed by staining cells with Annexin V-FITC Apoptosis Detection kit according to the manufacturer's instructions using flow cytometry (FACscalibur, Becton Dickinson, NJ). Fluorescence intensity was measured at the excitation wavelengths of 488 and 543 nm, respectively. Cells were treated the same as confocal imaging of apoptosis.

### 2.9. Statistical Analysis

Results obtained from 6 experiments are expressed as mean ± SD. Statistical significance was determined using one-way ANOVA followed by Tukey's test. *P* < 0.05 was considered as statistically significant.

## 3. Results

### 3.1. Effects of 2-DG on ERS and mPTP Opening

**Figure 1:**
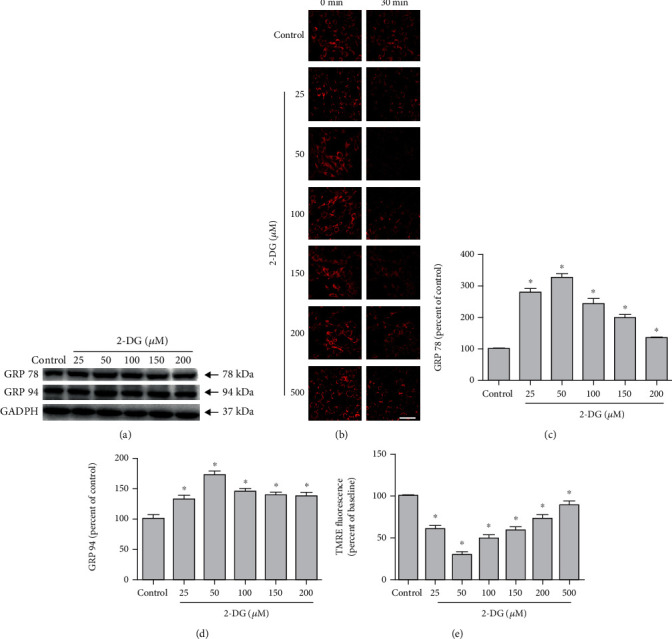
Effects of 2-DG on ERS and the mPTP opening. (a) Western blot images of GRP 78 and GRP 94 expression in PC12 cells treated with vehicle control and 25, 50, 100, 150, 200, and 500 *μ*M 2-DG. (b) Confocal fluorescence images of cells incubating TMRE for 20 minutes in PC12 cells treated with 0, 25, 50, 100, 150, 200, and 500 *μ*M 2-DG. (c, d) Quantification of (a). Each protein expression level was normalized with GAPDH. (e) Quantification of (b). Data are mean ± SD of 6 independent experiments. ^∗^*P* < 0.05 compared to vehicle control. Scale bar, 50 *μ*m.

PC12 cells were treated with different concentrations (0, 25, 50, 100, 150, and 200 *μ*M) of 2-DG to induce ERS. The expressions of GRP 78 and GRP 94 increased most significantly in cells treated with 50 *μ*M 2-DG (Figure 1(a)). Similarly, the intensity of TMRE red fluorescence decreases the most in cells treated with 2-DG at 50 *μ*M (Figure 1(b)). According to these results, we treated PC12 cells with 50 *μ*M 2-DG in the subsequent experiments.

### 3.2. Effect of Astragaloside IV on GSK-3*β* Phosphorylation

**Figure 2:**
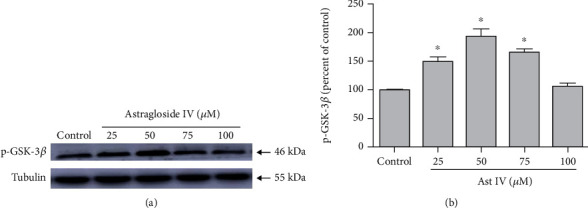
Effect of astragaloside IV on GSK-3*β* phosphorylation. (a) Western blot image of p-GSK-3*β* expression in PC12 cells treated with vehicle control and 25, 50, 75, and 100 *μ*M astragaloside IV. (b) Quantification of (a). Each protein expression level was normalized with Tubulin. Data are mean ± SD of 6 independent experiments.^∗^*P* < 0.05 compared to vehicle control.

PC12 cells were treated with astragaloside IV at different concentrations (25, 50, 75, and 100 *μ*M) to find out the concentration with the most neuroprotection effect. We found the expression of p-GSK-3*β* increased most significantly in cells treated with astragaloside IV at 50 *μ*M (Figure 2). Therefore, we use 50 *μ*M astragaloside IV in the subsequent experiments.

### 3.3. Effect of Astragaloside IV on PC12 Cell Survival from ERS

**Figure 3:**
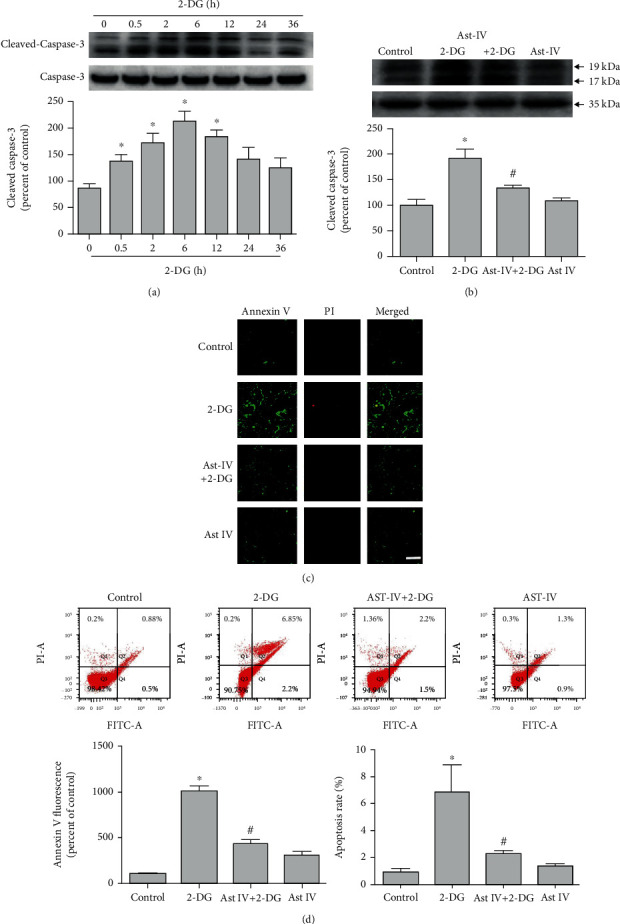
Effect of astragaloside IV on PC12 cell survival from ERS. (a) Representative Western blot images of Cleaved-Caspase-3 in PC12 cells treated with 2-DG in different times. (b) Representative Western blot images of Cleaved-Caspase-3 in PC12 cells treated with 2-DG and astragaloside IV. (c, d) Confocal fluorescence images and flow cytometry of Annexin V-FITC Apoptosis Detection kit in PC12 cells treated with 2-DG and astragaloside IV. Cells were preloaded with Annexin V and PI for 20 minutes before other treatments. PC12 cell culture was placed on the temperature-controlled stage and treated with astragaloside IV for 20 minutes and 2-DG for 6 h. Data are mean ± SD of 6 independent experiments. ^∗^*P* < 0.05 compared to control; ^#^*P* < 0.05 compared to 2-DG. Scale bar, 50 *μ*m.

2-DG in different times (0-36 hr) showed a significant increase of Cleaved-Caspase-3 with the peak at 6 hr (Figure 3(a)). 2-DG significantly reduced Cleaved-Caspase-3 expression (Figure 3(b)) and increased the green fluorescence intensity of Annexin V-FITC (Figure 3(c)) and apoptosis rate (Figure 3(d)); these effects were reversed by astragaloside IV, respectively, indicating that astragaloside IV protected PC12 cells from ERS-induced apoptosis.

### 3.4. Effect of Astragaloside IV on PC12 Cells from ERS

**Figure 4:**
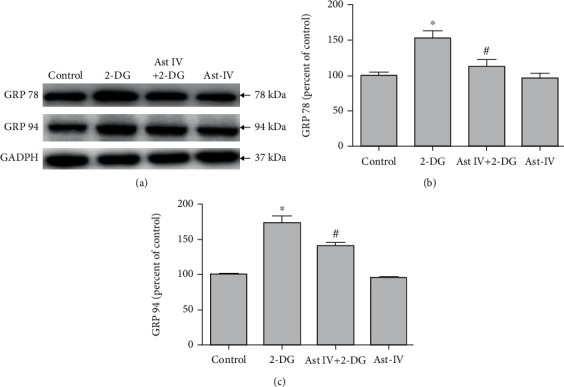
Effect of astragaloside IV on PC12 cells from ERS. (a) Western blot images of ER chaperone proteins GRP 78 and GRP 94 in PC12 cells treated with 2-DG and astragaloside IV. (b, c) Quantification of (a). Each protein expression level was normalized with GAPDH. Data are mean ± SD of 6 independent experiments. ^∗^*P* < 0.05 compared to vehicle control; ^#^*P* < 0.05 compared to 2-DG.

2-DG significantly increased GRP 78 and GRP 94 expressions while astragaloside IV reduced the increasement induced by 2-DG (Figure 4), indicating that astragaloside IV induced neuroprotection on PC12 cells by preventing ERS.

### 3.5. Effect of Astragaloside IV on ERS-Induced mPTP Opening

**Figure 5:**
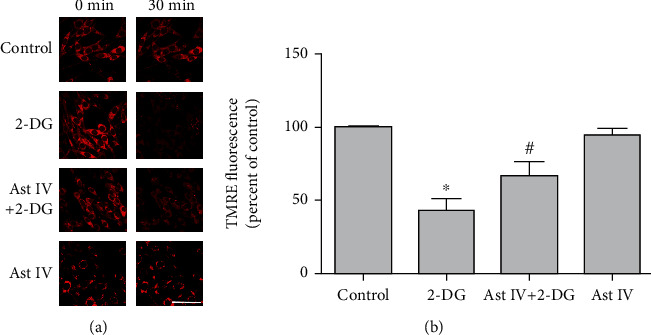
Effect of astragaloside IV on PC12 cells from ERS-induced mPTP opening. (a) Confocal images of TMRE probe in PC12 cells treated with 2-DG and astragaloside IV. Cells were preloaded with TMRE probe for 20 minutes before other treatments. PC12 cell culture was placed on the temperature-controlled stage and treated with astragaloside IV for 20 minutes and 2-DG for 30 minutes. (b) Quantification of TMRE Red fluorescence expressed as % of the control. The fluorescence intensity value was calculated as 30-minute normalized to 0-minute. Data are mean ± SD of 6 independent experiments. ^∗^*P* < 0.05 compared to control; ^#^*P* < 0.05 compared to 2-DG. Scale bar, 50 *μ*m.

We determined the ERS-induced loss of *ΔΨ*m by monitoring change in TMRE fluorescent intensity with confocal microscopy. 50 *μ*M of 2-DG induced a remarkable decrease in TMRE fluorescence in PC12 cells. Cells pretreated with 50 *μ*M astragaloside IV showed less reduction in TMRE fluorescence after 2-DG treatment (Figure 5), suggesting that astragaloside IV may protect PC12 cells by preventing the ERS-induced mPTP opening.

### 3.6. Effect of Astragaloside IV on ERS

**Figure 6:**
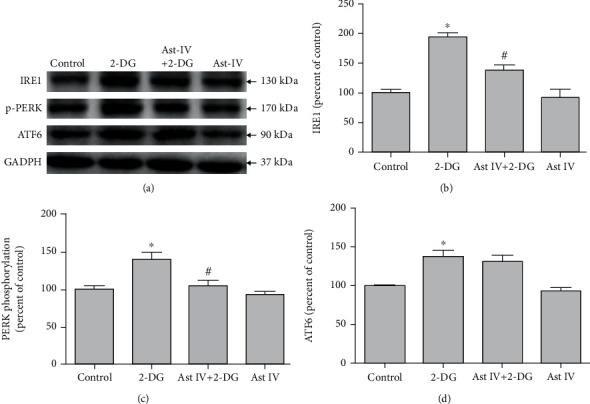
Effect of astragaloside IV on ERS signaling pathway. (a) Representative Western blot images of IRE1, p-PERK, and ATF6 in PC12 cells treated with 2-DG and astragaloside IV. (b–d) Quantification of (a). Each protein expression level was normalized with GAPDH. Data are mean ± SD for 6 independent experiments. ^∗^*P* < 0.05 compared to vehicle control; ^#^*P* < 0.05 compared to 2-DG.

We examined IRE1, p-PERK, and ATF6 expressions by western blotting. 2-DG significantly increased their expressions, while astragaloside IV significantly inhibited 2-DG-induced increasement of IRE1 and p-PERK, but not ATF6 (Figure 6). These results suggest that the IRE1/PERK but not the ATF6 signaling pathway is necessary for astragaloside IV to protect PC12 cells from ERS.

### 3.7. Effect of Astragaloside IV on GSK-3*β* Phosphorylation from ERS

**Figure 7:**
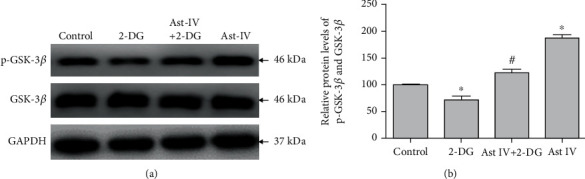
Effect of astragaloside IV on GSK-3*β* phosphorylation from ERS. (a) Representative Western blot image of p-GSK-3*β* in PC12 cells treated with 2-DG and astragaloside IV. (b) Quantification of (a). The relative level of p-GSK-3*β* was normalized with GAPDH and then to that of GSK-3*β*. Data are mean ± SD for 6 independent experiments. ^∗^*P* < 0.05 compared to vehicle control; ^#^*P* < 0.05 compared to 2-DG.

To determine the role of GSK-3*β* in the neuroprotection of astragaloside IV, we tested the expression of phosphorylated GSK-3*β*. Astragaloside IV significantly increased the phosphorylation of GSK-3*β* (Figure 7), suggesting that the inactivation of GSK-3*β* is necessary for astragaloside IV to protect PC12 cells from ERS.

## 4. Discussion

Stroke is one of the cerebrovascular diseases whose total mortality increases every year, with a high demand of neuroprotection. Ischemia-reperfusion injury (IRI) plays a key role in nerve damage caused by cerebral infarction [35, 36]. Endoplasmic reticulum stress-induced unfolded protein response is the main adaptive regulatory mechanism [37].

Nonmetabolizable glucose analogue 2-DG blocks glycolysis and glucose metabolism and inhibits protein glycosylation and ER quality control [38]. 2-DG significantly reduces ATP activity, inducing ERS in cells and inhibiting tumor growth and its anticancer or antiviral were tested in multiple studies [39, 40]. We found that 2-DG treatment significantly reduced the PC12 cell survival. We determined the effects of different concentrations (0, 25, 50, 100, 150, 200, and 500 *μ*M) of 2-DG treatment on the expressions of ERS chaperones GRP 78 and GRP 94 and found 50 *μ*M of 2-DG increased GRP 78 and GRP 94 expressions most significantly. In addition, we found 50 *μ*M of 2-DG decreased the red fluorescence of mitochondrial TMRE most significantly.

The main bioactive substance of Astragalus membranaceus bunge, a herb possessing protective activities for thousands of years [41], astragaloside IV demonstrates potent protective effect on focal cerebral ischemia/reperfusion [42], cardiovascular disease [43], pulmonary disease [44], liver fibrosis [45], and diabetic nephropathy [46] through several mechanisms including anti-inflammatory [47], antifibrotic [44], and antioxidative stress [48]. Our previous results have shown that 50 *μ*M of astragaloside IV treatment protects H9c2 cardiac cells by inhibition of GSK-3*β* and prevention of the mPTP opening [32].

Previous studies have shown that astragaloside IV has a neuroprotective effect on cortical neurons by inducing Nrf2 activation [9]. Astragaloside IV reduced glutamate-induced neurotoxicity and hypoxia-induced damage through the Raf-MEK-ERK pathway and miR-124 in PC12 cells [10, 11]. We examined the effect of different concentrations (0, 25, 50, 75, and 100 *μ*M) of astragaloside IV on GSK-3*β* phosphorylation in PC12 cells. Among different concentrations of astragaloside IV treatment, 50 *μ*M of astragaloside IV was most significant to induce GSK-3*β* phosphorylation.

The endoplasmic reticulum of eukaryotic cells is a membrane-bound organelle, performing various functions including synthesis of proteins to degradation of xenobiotics. Bioaccumulation of drugs/chemicals/xenobiotics in the cytosol can trigger ERS [49]. GRP 78 and GRP 94 are chaperone proteins for protein synthesis and termination in ERS. Previous studies show that Fluoxetine [14], sulforaphane [15], and Harpagide [16] protect neurons from hypoxia-reoxygenation-induced neuron apoptosis by reducing ERS. To investigate whether astragaloside IV could inhibit ERS to protect neuronal cells, we examined the expressions of ERS-related proteins. We found 2-DG treatment significantly increased the expressions of both GRP 78 and GRP 94 in PC12 cells, while astragaloside IV inhibited this increasement.

Mitochondria are the key organelles in eukaryotic cells and contribute to cellular stress responses. Misfunction of mitochondria plays a key role in ERS and nerve damage [50]. MPTP is a nonspecific pore opening in the mitochondrial inner membrane when ERS occurs. The opening of mPTP allows molecules smaller than 1.5 kDa, including protons, to enter mitochondria freely, affecting membrane potential and causing cell death [51]. Studies have shown that selective inhibition of mPTP prevents neurodegeneration in experimental multiple sclerosis [20]. Low temperature-induced neuroprotection is associated with a decrease in mitochondrial membrane potential [21]. Here, we showed that 2-DG significantly weakened the red fluorescence intensity of TMRE, while astragaloside IV pretreatment inhibited the decrease caused by 2-DG. These results suggested that astragaloside IV suppressed the ERS-induced mitochondrial membrane potential decrease, which prevents mPTP opening and protects PC12 cells.

Prolonged ERS induces cell apoptosis through PERK, ATF, and CHOP [52]. The mitochondrial pathway mediated the ERS-induced apoptosis [53, 54]. Our recent studies showed that resveratrol inhibited ERS-related apoptotic protein CHOP, Caspase12, and JNK expressions induced by 2-DG [55]. In the present study, we checked the neuroprotection effect of astragaloside IV on PC12 cells from 2-DG-induced apoptosis. We found 50 *μ*M of 2-DG treatment for 2-12 hr significantly increased Cleaved-Caspase-3 level with the peak at 6 hr. Astragaloside IV treatment inhibits PC12 cell apoptosis induced by 2-DG. In our study, we observed 2-DG significantly increased the Annexin V-FITC fluorescence in both confocal image and flow cytometry, these effects were significantly blocked by astragaloside IV. Therefore, these data suggested that chronic ERS caused neuronal damages in PC12 cells and astragaloside IV-induced neuroprotection via antiapoptosis. In our cell survival assay, we confirmed that 50 *μ*M of astragaloside IV is well tolerated in PC12 cell with minimal apoptosis detected (Figure 3(d)), and suggesting such a concentration is neuroprotective with low adverse effects.

Dysregulation of ER functions leads to the accumulation of misfolded-or unfolded-protein in the ER lumen and UPR. IRE1, PERK, and ATF6 are three UPR downstream pathways regulating the gene expressions to maintain ER homeostasis [56]. Unmitigated ERS leads to cell apoptosis [57]. Studies have shown that the PERK pathway plays a neuroprotective role in the brain injury caused by experimental cerebral hemorrhage [31]. Notoginsenoside R1 regulates ATF6/Akt pathway through estrogen receptor and reduces OGD/R-induced neuronal damage. RACK1 upregulation induces neuroprotection by activating the IRE1-XBP1 pathway in rat traumatic brain injury [58]. We found that 2-DG significantly increased the expressions of IRE1, p-PERK, and ATF6, while astragaloside IV inhibited the increases of IRE1 and p-PERK, but not ATF6. These data suggested that IRE1 and PERK pathways are involved in the neuroprotection by astragaloside IV.

GSK-3*β* kinase is a key signaling molecule regulating structural and functional synaptic plasticity in the normal brain [25]. Furthermore, the prevention of mPTP opening induced by GSK-3*β* phosphorylation is important to reduce cell damage. Grape seed-derived proanthocyanidins reduce neuronal oxidative damage by inhibiting the GSK-3*β*-dependent mPTP opening [18]. We found 2-DG significantly reduced GSK-3*β* phosphorylation, while astragaloside IV inhibited such phosphorylation. Our data suggested that astragaloside IV protects PC12 cells through GSK-3*β* phosphorylation.

**Figure 8:**
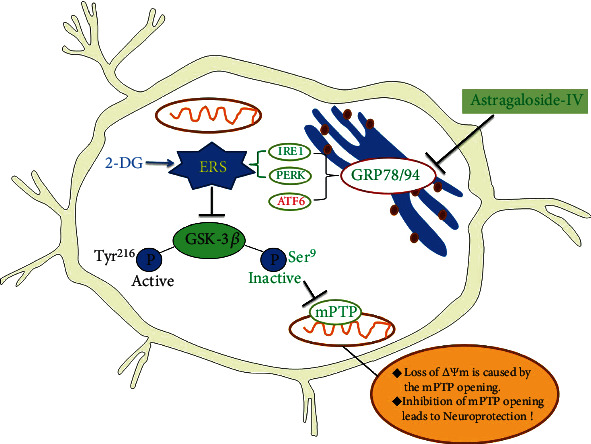
Mechanism of astragaloside IV-mediated neuroprotection on PC12 cells from ERS. Astragaloside IV protects PC12 cells from ERS by inactivation of GSK-3*β* and preventing the mPTP opening. The GRP 78, GRP 94, IRE1, and PERK signaling pathways but not ATF6 are responsible for GSK-3*β* inactivation and neuroprotection by astragaloside IV.

In conclusion, astragaloside IV protected PC12 cell survival from ERS by inactivating GSK-3*β* and preventing mPTP opening. We further show that IRE1 and PERK but not ATF6 is important for neuroprotection of astragaloside IV (Figure 8). Our *in vitro* results warrant the further study of neuroprotective mechanisms of astragaloside IV using *in vivo* models.

## Data Availability

The data used to support the findings of this study are included within the article.
